# Carvacrol potentiates immunity and sorafenib anti-cancer efficacy by targeting HIF-1α/STAT3/ FGL1 pathway: in silico and in vivo study

**DOI:** 10.1007/s00210-024-03530-9

**Published:** 2024-10-28

**Authors:** Eman H. Yousef, Amal M. El Gayar, Nada F. Abo El-Magd

**Affiliations:** 1 Biochemistry Department, Faculty of Pharmacy, Mansoura University, Mansoura, 35516 Egypt; 2 Department of Pharmacology and Biochemistry, Faculty of Pharmacy, Horus University-Egypt, New Damietta, 34518 Egypt

**Keywords:** Liver cancer, Tumor immunity, Drug resistance, Hypoxia, Hepassocin, Tumor microenvironment

## Abstract

Hypoxia and tumor cell immunological escape greatly hinder the hepatocellular carcinoma (HCC) treatment efficiency. This study is designed to investigate the capability of carvacrol (CVR) to enhance sorafenib (SOR) anti-cancer efficacy and modulate anti-HCC immunity. CVR target and biological activities were predicted using Swiss Target Prediction website and PASS web server. UALCAN and LinkedOmics databases were used to examine hypoxia-inducible factor 1-alpha (HIF-1α) expression and the relationship between studied genes and tumor clinical features. Kaplan–Meier plotter (KM plotter) and TISIDB databases were used to illustrate correlation of HIF-1α with HCC prognosis and immune infiltration. The binding affinities of CVR to p300, KAT2B, CREBBP, and Hsp90 were demonstrated by molecular docking. In vivo analysis was performed in male Sprague–Dawley rats. The STAT3, JAK2, and fibrinogen-like protein 1 (FGL1) expressions were assessed by qRT-PCR. FGL1 was determined by ELISA. CD8^+^ T cell number was counted by flow cytometry. HIF-1α was determined by immunohistochemistry. CVR showed an HIF-1α inhibitory potential, which is highly expressed in HCC tissues. Also, elevated HIF-1α expression has been found to be correlated with clinicopathological characteristics, poor survival in HCC patients, and tumor immune cell infiltration. CVR/SOR enhanced liver functions and decreased AFP level. CVR/SOR hindered HCC progression by downregulating STAT3, JAK2, and FGL1. CVR/SOR induced tumor immunity via increasing CD8^+^ T cells. CVR/SOR is a powerful combination for tumor repression and enhancing SOR efficiency in HCC by modulating FGL1. Moreover, CVR/SOR might exert the aforementioned effects through HIF-1α/STAT3/FGL1 pathway.

**Supplementary Information:**

The online version contains supplementary material available at 10.1007/s00210-024-03530-9.

## Introduction

Hepatocellular carcinoma (HCC), with more than 500,000 newly diagnosed cases per year, is the 5th most frequent malignancy in the world (Wen et al. 2022). Even through current therapeutic approaches such as surgical resection, ablation, liver transplantation, and monoclonal antibody therapy, 5-year survival represents only 18% due to late diagnosis (Yarchoan et al. 2019). Sorafenib (SOR) is a multi-kinase inhibitor used to treat advanced HCC by attenuating cell proliferation and blocking tumor angiogenesis (Wang et al. 2022b). Despite SOR treatment has stated to increase median survival by about 3 months (Chen et al. 2016), its efficacy is limited due to hypoxia and immune microenvironment (Wang et al. 2022b).

Hypoxia is a main feature of all solid tumors (Lequeux et al. 2021). In response to hypoxia, tumor cells adjust their metabolism and growth to low-oxygen circumstances. Hypoxia-inducible factors (HIFs) and HIF-1 signaling pathways have a significant impact on these hypoxic responses. HIF-1α nuclear translocation is essential for HIF-1 activation (Chavda et al. 2022). Under normoxic condition, prolyl-4-hydroxylase (PHD) hydroxylates two conserved proline residues in HIF-1α, enabling it to interact with the E3 ligase von Hippel-Lindau (VHL) protein and be degraded by the ubiquitin–proteasome system (Li et al. 2019). Additionally, factor inhibiting HIF-1 (FIH) prevents HIF-1 from interacting with coactivator p300/CBP, thereby hindering HIF-1α activity (Ikeda and Kakeya 2021). Contrarily, under hypoxic circumstances, both PHD and FIH decline (Ikeda and Kakeya 2021). KAT2B acetylates HIF-1α enhancing its interaction with p300 (Waddell et al. 2021). Also, newly synthesized HIF-1α is stabilized by the chaperone heat shock protein 90 (HSP90) through conformational changes in its structure (Kataria et al. 2019a, b). As a result, rather than its degradation, it accumulates and translocates to the nucleus, where it forms heterodimer with HIF-1β. When this heterodimer with p300/CBP binds to the hypoxia response element (HRE), it induces the expression of a large number of genes that are related to several biological processes, including erythropoiesis, pH regulation, anaerobic glycolytic metabolism, angiogenesis, inflammation and immunity, cell proliferation and survival, and cancer metastasis (Yeo 2019; Ikeda and Kakeya 2021).

Carvacrol (CVR) is a natural-bioactive monoterpenoid phenolic compound which has been approved by the FDA as a chemical flavoring for food use (Heidarian and Keloushadi 2019). CVR is extracted from many aromatic plants that are usually used for therapy/prevention purposes in folk medicine (Bayir et al. 2019). It has been well investigated for its various biological activities, including anti-inflammatory (Gunes-Bayir et al. 2022), anxiolytic (Pires et al. 2013), analgesic (Horishita et al. 2020), antiparasitic (Marjanović et al. 2020), and neuroprotective (Yıldız et al. 2022) properties. Furthermore, CVR has antiproliferative effects on various cancer types, such as prostate (Khan et al. 2019), colon (Pakdemirli et al. 2020), lung (Alanazi et al. 2022), liver (Ahmed et al. 2015), breast (Kapetana et al. 2022), cervical (Ahmad and Ansari 2021), and neuroblastoma (Chenet et al. 2019). Sivaranjani et al. (2016) have found that CVR has chemopreventive effect against 1,2-dimethylhydrazine–induced experimental colon carcinogenesis (Sivaranjani et al. 2016). Also, Jamali et al. (2020) have reported that *Oliveria decumbens* Vent. essential oil, containing 23.12% CVR, exerts antitumor efficacy against 4T1 tumor in vivo by modulating host immune response (Jamali et al. 2020). In our previous study, we explored the CVR antitumor effect through TRPM7 inhibition (Yousef et al. 2023a). In the current study, we will explore more the underlying mechanism through in silico and in vivo study.

## Materials and methods

### Target and biological activity predictions

Studies of molecular target are crucial to identify the side effects of phenotypical or possible cross-reactivity induced by the small biomolecule action. Firstly, the ZINC number for CVR (ZINC967563) was inserted into the search field on the Swiss Target Prediction website (https://www.swisstargetprediction.ch). The PASS web server was then used to predict the CVR’s possible biological activities based on their chemical composition by using multilevel neighbors of atom (MNA) descriptors. The probability of being active (Pa) and the probability of being inactive (Pi) are used to calculate the biological activity prediction score. A higher Pa indicates that the molecule is more likely to exhibit the biological feature (Jairajpuri et al. 2021).

### UALCAN and LinkedOmics analysis

The comprehensive online site Ualcan (http://ualcan.path.uab.edu/) is used to analyze cancer OMICS data. Our study analyzed the expression of HIF-1α in HCC based on patient characteristics such as gender, age, race, and TP53 mutation status as well as histological subtypes and specific disease stages. Transcripts per million readings were used to normalize the expression level of HIF-1α. *P* < 0.05 was considered statistically significant. Additionally, LinkedOmics (http://www.linkedomics.org) database was used to analyze the TCGA clinical data in order to explore the relationship between studied genes and tumor clinical characteristics.

### Kaplan–Meier (KM) plotter database

The KM plotter (http://kmplot.com) database was used to examine prognostic values for HIF-1α expression in HCC, including overall survival (OS), relapse-free survival (RFS), progression-free survival (PFS), and disease-specific survival (DSS) based on Affymetrix microarrays.

### Docking of CVR to p300, KAT2B, CREBBP, and Hsp90

As HIF-1α is not a druggable structure, the docking study cannot be conducted on it. Instead, we chose the other proteins “p300,” “KAT2B,” “CREBBP,” and “HSP90” which are involved in HIF-1α activation and preventing its degradation under hypoxic circumstances for CVR molecular interaction study. The crystal structures of p300 (PDB ID: 3BIY), KAT2B (PDB ID: 5FE7), CREBBP (PDB ID: 4NR7), and Hsp90 (PDB ID: 2xjx) were retrieved from the Protein Data Bank (https://www.rcsb.org/pdb/home/home.do). In each protein, water molecules and complexes bound to the protein were deleted. Moreover, the ionization and tautomeric states of the amino acid residues were adjusted by adding hydrogens to the protein. A binding active site is identified for each protein based on the documented ligand/protein interactions. The ZINC database was used to extract the three-dimensional configuration of CVR, and its chemical structure optimization was carried out by modifying the bond order, adding charges, and finally adding hydrogens. Additionally, energy minimization by conformational search or at least clean-up geometry was applied. Before adding hydrogens, bond order and charges were adjusted. To facilitate readability in the AutoDock software, the protein and ligand structure were converted into the PDBQT file format. Molecular docking was carried out using AutoDock Vina. The docking estimations were established in relation to the X, Y, and Z dimensions in the used grid map as indicated in Supplementary 1. Chimera 1.15 and Discovery Studio Visualizer v21.1.0.20298 were used to visualize and analyze the binding mode and interactions in the binding pocket of the obtained poses.

### DAVID functional enrichment analyses of studied genes

The DAVID database version 6.8 (https://david.ncifcrf.gov/) was used to integrate biological data and analytical tools for functional annotation of genes and pathways.

### Drugs and chemicals

Thioacetamide (TAA) with a purity of 99% and CVR were ordered from Sigma Aldrich Chemicals Co. (St. Louise, MO, USA). SOR was ordered from the market (Cipla, India). All other chemicals such as carboxymethyl cellulose (CMC) are of high analytical grade and were ordered from Elgomhoria Co. (Mansoura, Egypt). TAA was dissolved (10% w/v) in normal saline, while CVR and SOR were suspended (1% w/v) in 0.5% CMC.

### HCC model establishment

All animals received care based on the guidelines of the Animal Research Ethics Committee of the Mansoura University, Egypt, which came consistent with the guidelines for Care and Use of Laboratory Animals described by the US National Institutes of Health (NIH Publication, No. 85–23, revised 1985). Seventy-five males Sprague–Dawley weighing 190–250 g were purchased from “Egyptian Organization for Biological Products and Vaccines,” Giza, Egypt. Rats were kept for acclimation under standard laboratory conditions of controlled room temperature 25 ± 2 °C, with regular 12 h light/12 h dark cycle and allowed free access to food and water. The experimental HCC model has been established in rats by twice weekly i.p. injections of 200 mg/kg body weight thioacetamide (TAA) for 16 consecutive weeks (Helmy et al. 2019; Mohamed et al. 2022). The Success of the HCC model was confirmed by liver histopathological evaluation analysis and the significant elevation in serum ALT, AST, and alpha fetoprotein (AFP) levels in the HCC group as compared with the CMC group, which was consistent with our previous results (Yousef et al. 2023a).

### Experimental design

**Fig. 1:**
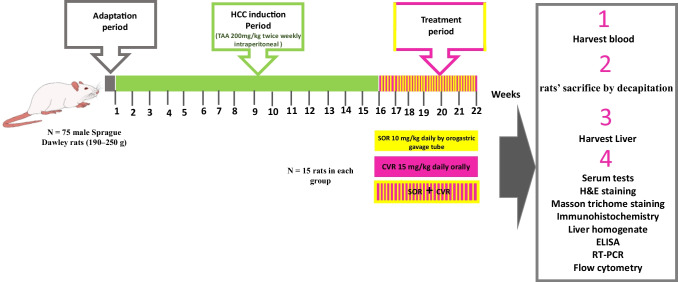
Experimental design. Rats were given 200 mg/kg of thioacetamide (TAA) intraperitoneally twice a week to establish a hepatocellular carcinoma (HCC) model. Following a 16-week treatment period, carvacrol (CVR) (15 mg/kg daily orally), sorafenib (SOR) (10 mg/kg daily orally), or a combination of both were given for 6 weeks. Blood and liver were collected to be used in other experiments. ELISA, enzyme-linked immunosorbent assay; H&E, hematoxylin and eosin; qRTPCR, quantitative reverse transcription polymerase chain reaction

The experimental design is clarified in Fig. 1. The grouping and regimens of treatment are the same as mentioned in our previously published work (Yousef et al. 2023a). Rats were randomly divided into five groups (*n* = 15) as follows: control group (CMC) in which rats were normal and injected with 0.9% normal saline intraperitoneally (i.p.) twice weekly for 16 weeks. Then, rats were gavaged CMC by intragastic tube daily for 6 weeks; HCC group in which rats had HCC but received no further treatment; SOR group in which rats had HCC then received 10 mg/kg SOR by intragastic tube for 6 weeks daily; CVR group in which rats had HCC then received 15 mg/kg CVR by intragastic tube for 6 weeks daily; CVR/SOR group in which rats had HCC then received both SOR and CVR by intragastic tube with the same mentioned doses for 6 weeks daily.

### Collection of biological samples

By the end of treatment, rats were weighed, fasted for the night, and then given pentobarbital sodium with a dose of 40 mg/kg, intraperitoneal to induce anesthesia. Before rats’ sacrifice by decapitation, blood samples were obtained through the retroorbital vein of the eye using a sterile, non-heparinized capillary tube to separate the serum. The liver was removed, rinsed with ice-cold 0.01 M phosphate buffer saline (PBS) (pH 7.4), dried, weighed, and divided into three pieces. To evaluate ELISA studies, one section was homogenized in 10% weight/vol, ice-cold, 0.01 M PBS (pH 7.4), separated into aliquots, and frozen at − 80 °C. For histopathological and immunohistochemical analysis, another section was immersed in 10% buffered formalin. For quantitative reverse transcription polymerase chain reaction (qRTPCR) and flow cytometry analyses, the final section of the liver was directly embedded in liquid nitrogen.

### The liver enzymes assessment

Serum was used to assess liver functions by measuring alanine aminotransferase (ALT) and aspartate aminotransferase (AST) activities (BioMed, Helioplise, Egypt) using the commercially available kits.

### ELISA assay

Hepatic fibrinogen-like protein 1 **(**FGL1) in liver homogenate and AFP in serum were determined quantitatively using enzyme-linked immunosorbent assay (ELISA) technique based on manufacturer’s instructions (Innova Biotech Co., Beijing, China).

### Histopathological investigation of liver tissues

Liver tissues were first fixed for 24 h in 10% buffered formalin, then washed with tap water, dehydrated with serial dilutions of alcohol, cleaned with xylene, and lastly embedded in paraffin blocks in a hot air oven at 56 °C for 24 h. Using a microtome, 5-µm-thick sections of paraffin blocks were cut. The resulting sections were collected on glass slides, deparaffinized, and stained with hematoxylin and eosin (H&E) stain for evaluation under a microscope. A digital camera-aided computer system (Nikon Digital Camera, Japan) was used to record and photograph histopathological alterations.

### Immunohistochemical analysis of HIF-1α expression

Immunohistochemical methods were used to assess HIF-1α expression, and HIF-1α antibody (Cat. No. A16873; ABclonal Co.) was used to stain parallel liver tissue sections.

### Flow cytometric analysis of CD8^+^ T cell

Single-cell suspensions were obtained by cutting the liver into small pieces and passing via a nylon mesh to discard cell aggregates. Cells were incubated with 10 µl of conjugated anti-CD8 antibody (Abbkines, California, CA, USA) at 4 °C in the dark for 30 min. Following two rounds of washing with 2 ml PBS containing 0.1% sodium azide (NaN3) and 1% BSA, the cells were centrifuged for 5 min at 1500 rpm. Cells were then put back together in 0.2 ml of 0.5% paraformaldehyde in PBS/BSA. Finally, a Becton Dickinson AccuriTM C6 flow cytometer was used to analyze CD8^+^ T cell populations. Data was also examined by BD Accuri C6 Software (Di Marzio et al. 1999).

### Quantitative real-time polymerase chain reaction (qRT-PCR)

According to the manufacturer’s recommendations, RNA was extracted from liver tissues using the RNeasy Mini extraction kit (Qiagen, Germany). The concentration and purity of the extracted RNA were measured spectrophotometrically by NanoDrop System (Thermo Fisher Scientific Inc., TX, USA) at 260 nm and 260/280 nm ratio, respectively. After that, SensiFAST™ cDNA Synthesis Kit (Bioline, USA) was used to reverse transcript 1 µg of RNA to cDNA. On an Azure Biosystems qPCR equipment, real-time PCR was performed using TOPrealTM SYBR Green qPCR 2X PreMIX (SYBR green with low ROX) in accordance with the manufacturer’s instructions. The previously established gene-specific PCR primers are used (Yousef et al. 2023b) and illustrated in Supplementary 1. The mRNA expressions of these genes were normalized against rat glyceraldehyde 3-phosphate dehydrogenase (GAPDH) as a housekeeping gene and determined using the 2^−ΔΔCt^ method.

### Statistical analysis

Mean ± standard error of mean (SEM) was used to represent all data. A one-way analysis of variance (ANOVA) and Tukey’s test were used to assess the statistical significances. Any *P*-value that was 0.05 or below was regarded as significant. To investigate correlations between variables, Pearson’s correlation coefficients (*R*) were calculated. The SPSS program, version 16.0 (SPSS, Inc., Chicago, IL, USA), was applied for all analyses. Graph Pad Prism 6.01 (Graph Pad Software, San Diego, CA, USA) was used to create the graphs.

## Results

### Target and biological activity predictions

**Fig. 2:**
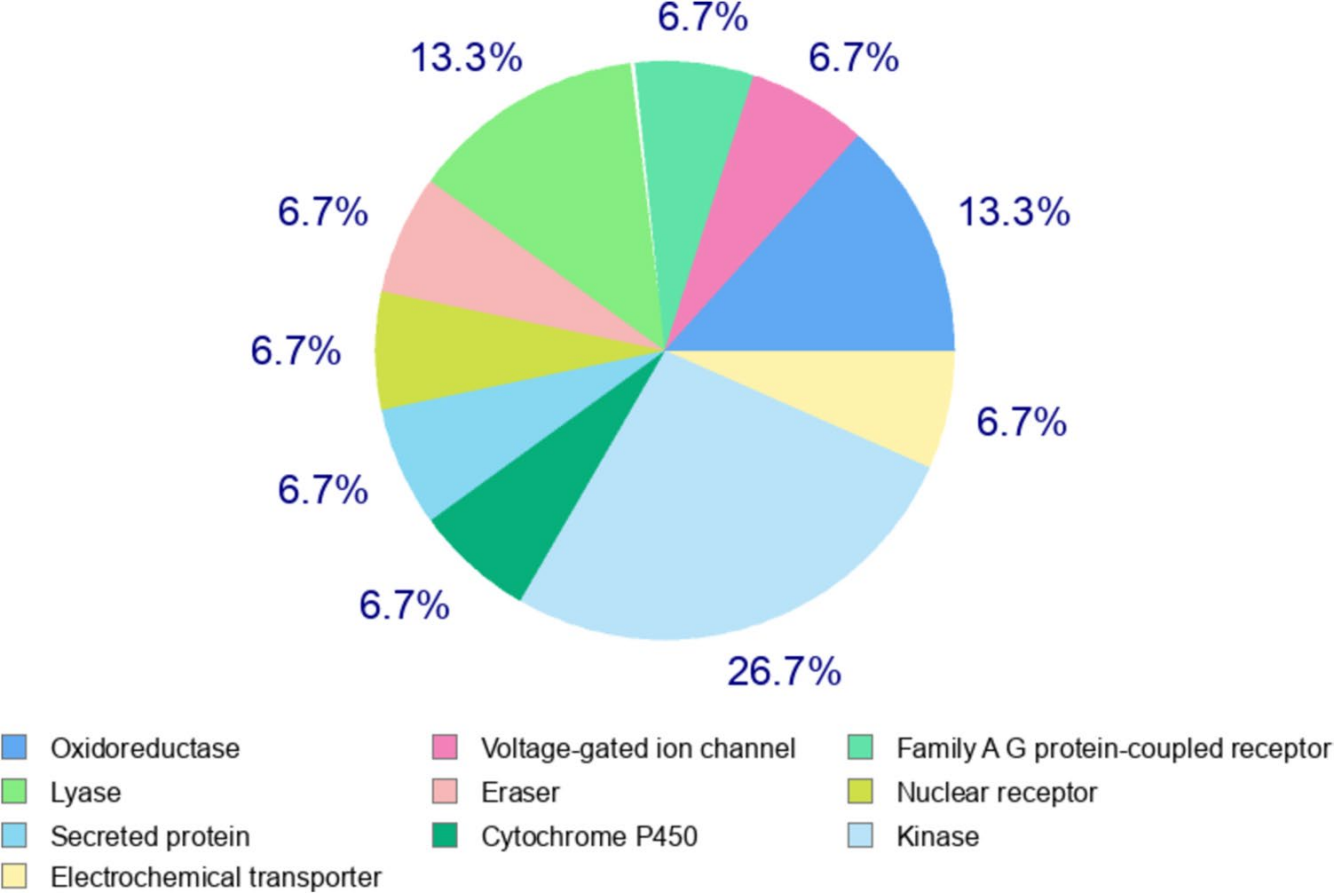
Top ten targets predicted for carvacrol (CVR) using the Swiss Target Prediction website. The pie chart represents that among the feasible targets of CVR are kinases, oxidoreductase, lyases, secreted proteins, voltage-gated ion channels, family AG protein-coupled receptors, erasers, nuclear receptors, electrochemical transporters, and cytochrome P450

The top ten findings of target prediction analysis were presented as a pie chart. The pie chart represents that among the feasible targets, 26.7% are kinases, 13.3% belong to the oxidoreductase, 13.3% are lyases, 6.7% are secreted proteins, 6.7% are voltage-gated ion channels, 6.7% are family AG protein-coupled receptors, 6.7% are erasers, 6.7% are nuclear receptors, 6.7% are electrochemical transporters, and 6.7% are cytochrome P450 (Fig. 2). The outcome of target prediction consisting of target, common name, Uniprot ID, ChEMBL-ID, target class, probability, and known actives in 2 D/3D is displayed in Supplementary 2. It has been found that CVR targets JAK2 with probability score 0.0439. Furthermore, the PASS analysis of the CVR’s biological activities revealed comparable types of biological activities. CVR was shown to have HIF-1, JAK2, MMP9, HSP90, and STAT3 inhibitory activities. The biological properties of CVR with increased Pa are shown in Table 1.

**Table 1:** List of biological properties of carvacrol (CVR) calculated through PASS webserver

Compound	Biological activity	Pa	Pi
**CVR**	HIF-1α expression inhibitor	0.864	0.008
	JAK2 expression inhibitor	0.737	0.014
	Anti-inflammatory	0.674	0.019
	MMP9 expression inhibitor	0.652	0.01
	TP53 expression enhancer	0.57	0.06
	Heat shock protein 90 antagonist	0.211	0.004
	Transcription factor STAT3 inhibitor	0.197	0.134

*HIF-1α* hypoxia-inducible factor-1 apha, *JAK2* Janus kinase 2, *MMP9* matrix metallopeptidase 9, *Pa* probability to be active, *Pi* probability to be inactive, *STAT3* signal transducers and activators of transduction, *TP53* tumor protein p53

### Elevated HIF-1α expression is correlated with clinicopathological characteristics and poor survival in HCC patients

**Fig. 3:**
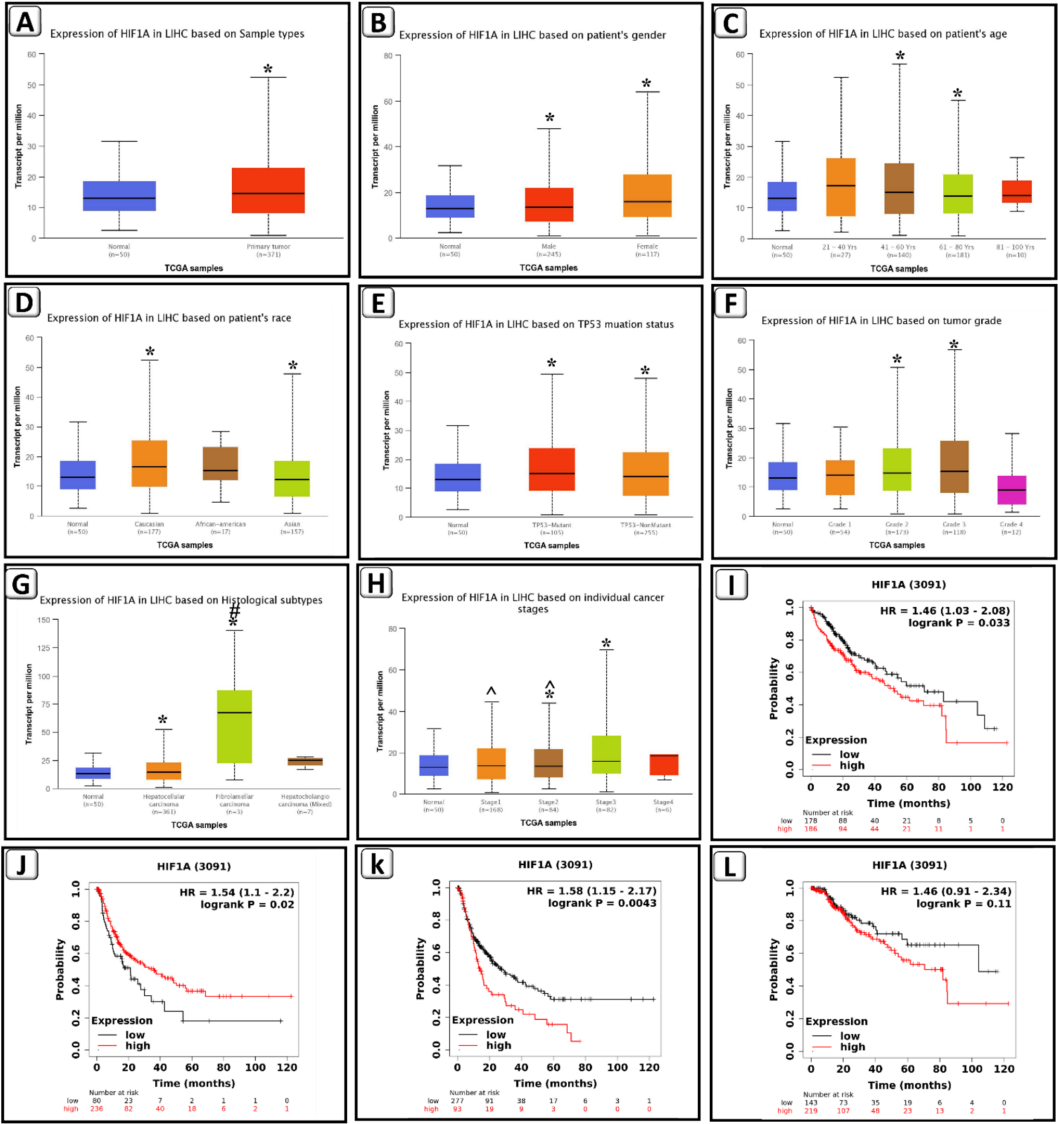
Elevated hypoxia-inducible factor 1-alpha (HIF-1α) expression is correlated with clinicopathological characteristics and poor survival in hepatocellular carcinoma (HCC) patients. **A** Boxplot shows expression of HIF-1α in liver cancer. **B** HIF-1α expression based on patient’s gender in HCC. **C** HIF-1α expression based on patient’s age in HCC. **D** HIF-1α expression based on patient’s race in HCC. **E** HIF-1α expression based on Tp53 mutation status in HCC. **F** HIF-1α expression based on tumor grade in HCC. **G** HIF-1α expression based on histological subtypes in HCC. **H** HIF-1α expression based on cancer stages in HCC. **I** Overall survival. **J** Relapse-free survival. **K** Progression-free survival. **L** Disease-specific survival. For all the analysis, *P* < 0.05 was considered statistically significant. *Significance against normal; #significance against HCC; ^significance against stage 3 HCC

The expression of the HIF-1α gene in HCC tissues (371 cases) and normal tissues (50 cases) was analyzed using TCGA data in the UALCAN online tool. HIF-1α expression was high in HCC tissues, as seen in Fig. 3, and the differences were statistically significant. HIF-1α expression was not related to patients’ gender, age, race, or TP53 mutation status (*P* > 0.05). On the other hand, high HIF-1α expression was significantly linked with histological subtypes and individual cancer stages (*P* < 0.05).

Furthermore, the Kaplan–Meier plotter database was utilized to correlate HIF-1α mRNA level with OS, RFS, and PFS in HCC patients. HCC patients with elevated HIF-1α expression showed worse OS and PFS and higher RFS than those with low HIF-1α expression (Fig. 3).

Moreover, the results of LinkedOmics demonstrated that HIF-1α was significantly linked with overall survival, race, and histological type, indicating that high expression of HIF-1α gene may have critical roles in various ethnic populations, as well as predict poor survival and tumor progression, while STAT3 in HCC patients was significantly correlated with race. Also, FGL1 was significantly correlated with pathologic stage and histological type, revealing that elevated expression of FGL1 gene predicted tumor progression (Table 2).

**Table 2:** Relationship between selected upregulated genes and clinical features in HCC

Item	*N*	HIF-1α statistic	*P*-value	STAT3 statistic	*P*-value	JAK2 statistic	*P*-value	FGL1 statistic	*P*-value
Overall survival (Cox regression test)	343 ≤ median 172 > median 171	0.1943	**0.02108**	0.0291	0.3148	0.052	0.5812	0.00755	0.3333
Pathologic stage (Kruskal–Wallis test)	347 Stage I 171 Stage II 86 Stage III 85 Stage IV 5	0.09174	0.09174	0.01140	0.08293	0.04148	0.4556	0.0158	**0.03284**
Race (Kruskal–Wallis test)	(361) American Indian or Alaska Native 2 Asian 158 Black African American 17 White 184	10.21	**0.01690**	13.15	**0.004317**	6.1	0.1069	3.648	0.3021
Histological type (Kruskal–Wallis test)	Fibrolamellar carcinoma 3 Hepatocellular carcinoma 361 Hepatocholangiocarcinoma 7	7.830	**0.01994**	1.881	0.3903	4.491	0.1059	6.308	**0.04268**

*FGL1* fibrinogen-like protein 1, *HIF-1α* hypoxia-inducible factor-1 alpha, *JAK2* Janus kinase 2, *STAT3* signal transducers and activators of transduction. Bold indicates *p* value less than 0.05

### CVR molecular interaction study

**Fig. 4:**
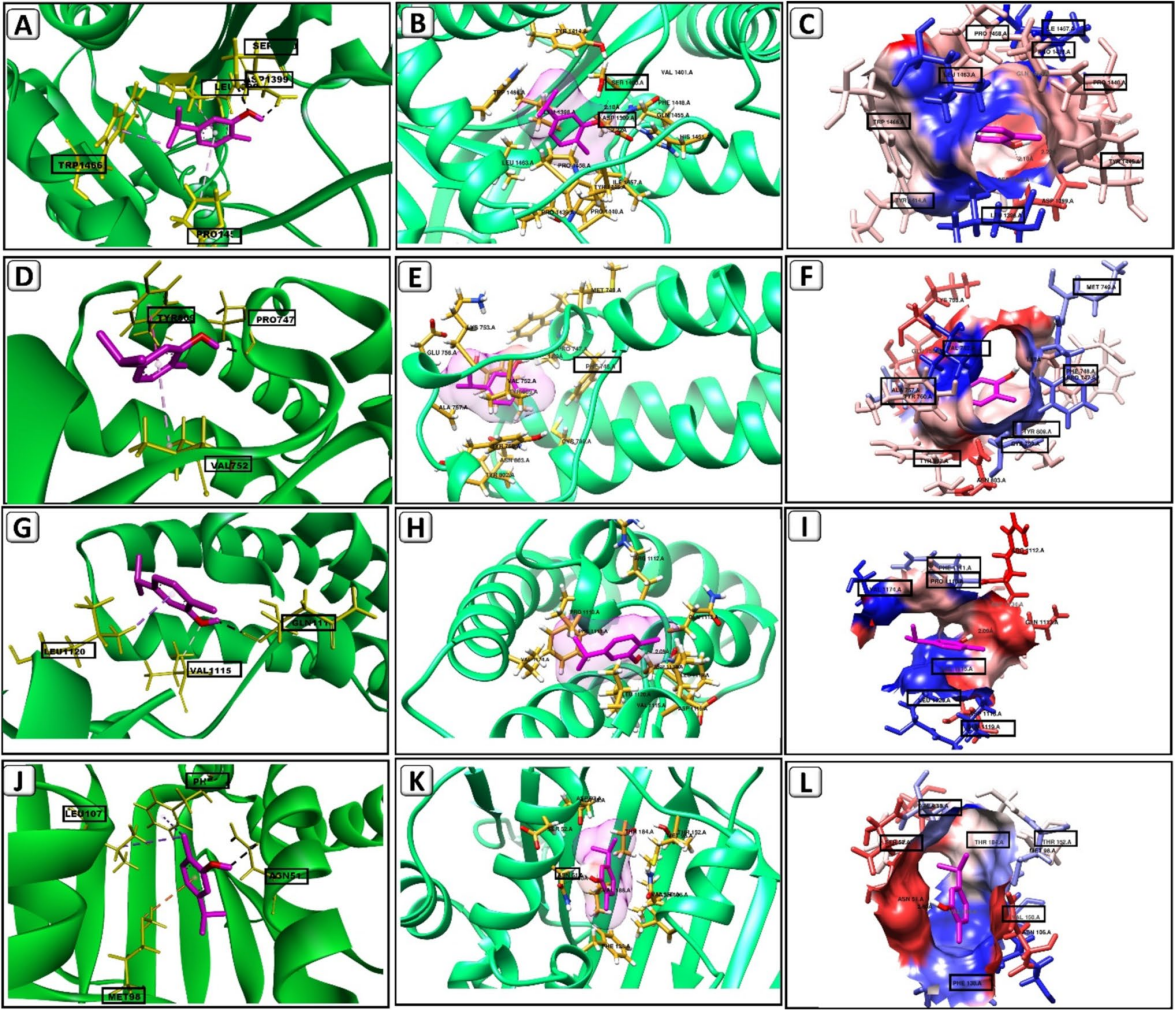
Docked pose of CVR in the binding pocket of p300, KAT2B, CREBBP, and HSP90. **A** Amino acids participate in the p300 interaction using Discovery Studio Visualizer. **B** Hydrogen bond between CVR and p300 pocket using Chimera. **C** Hydrophobic interactions between CVR and p300 pocket using Chimera. **D** Participating amino acids in the interaction of KAT2B using Discovery Studio Visualizer. **E** Hydrogen bond between CVR and KAT2B pocket using Chimera. **F** Hydrophobic interactions between CVR and KAT2B pocket using Chimera. **G** Participating amino acids in the interaction of CREBBP using Discovery Studio Visualizer. **H** Hydrogen bond between CVR and CREBBP pocket using Chimera. **I** Hydrophobic interactions between CVR and CREBBP pocket using Chimera. **J** Participating amino acids in the interaction of HSP90 using Discovery Studio Visualizer. **K** Hydrogen bond between CVR and HSP90 pocket using Chimera. **L** Hydrophobic interactions between CVR and HSP90 pocket using Chimera. CREBBP, CREB binding protein; CVR, carvacrol; HSP90, heat shock protein 90; KAT2B, lysine acetyltransferase 2B; p300, histone acetyltransferase

**Fig. 5:**
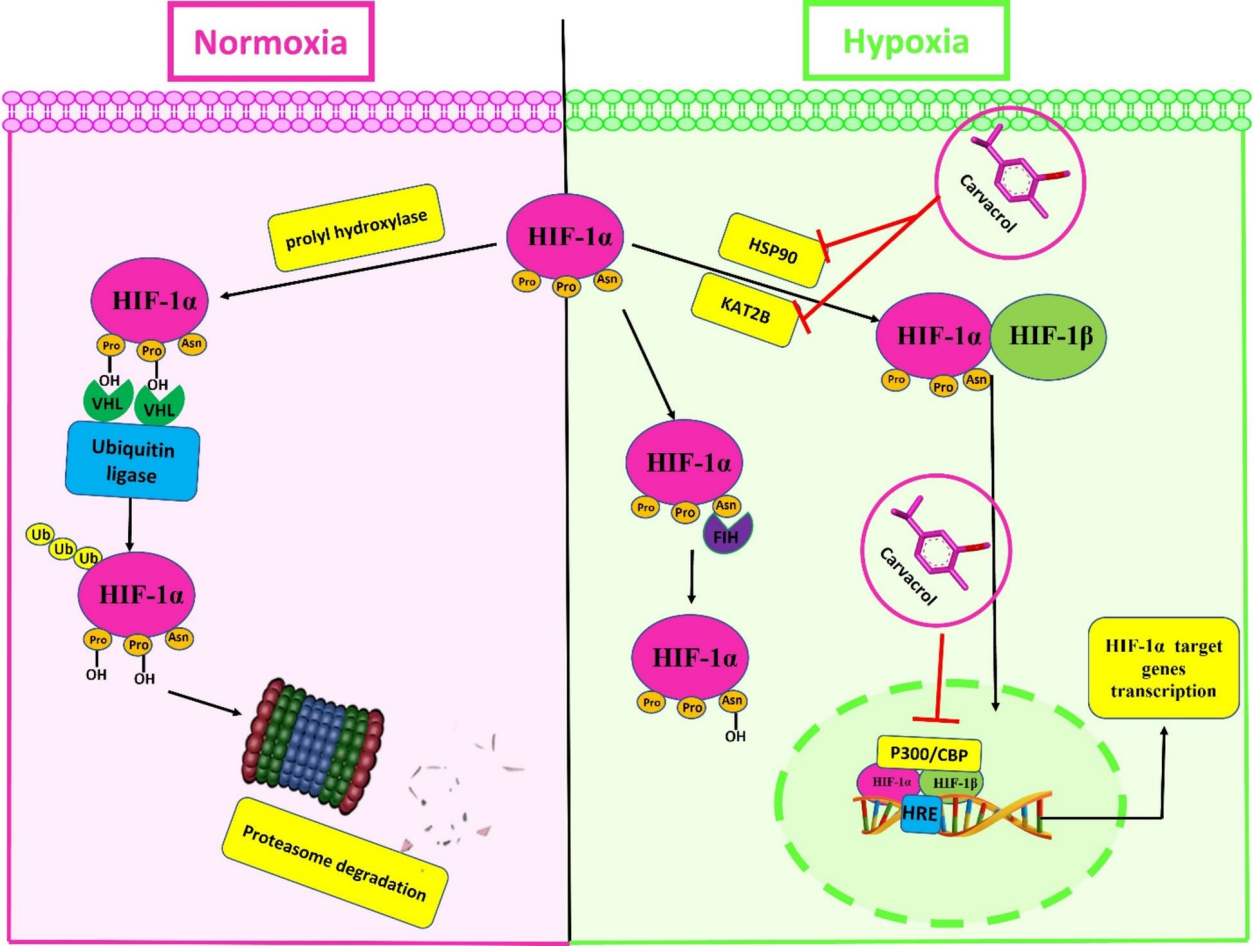
The proposed mechanism of carvacrol (CVR) according to docking results. CVR inhibits hypoxia-inducible factor-1 alpha (HIF-1α) signaling activation through inhibiting HSP90, KAT2B, and P300/CBP. CBP, CREB binding protein; HRE, hypoxia response element; HSP90, heat shock protein 90; KAT2B, lysine acetyltransferase 2B; p300, histone acetyltransferase

CVR/p300 binding interactions were estimated using AutoDock Vina 1.1.2 (Trott and Olson 2010). The binding affinity was − 6.3 kcal/mol when CVR interacted with the p300. A two-hydrogen bond was established between CVR with Ser1400 and Asp1399 of p300. The binding of CVR with p300 is promoted by numerous hydrophobic interactions with Leu1398, Leu1463, Ile1457, Typ1446, Trp1466, Tyr1414, Pro1458, Pro1439, and Pro1440 of p300. The binding affinity was − 6.2 kcal/mol when CVR interacted with the KAT2B. A hydrogen bond was established between CVR with Pro747 of KAT2B. The CVR/KAT2B binding is promoted by numerous hydrophobic interactions such as with Val752, Phe748, Cys799, Met749, Ala757, Trp746, Tyr809, Tyr802, and Tyr760 of KAT2B.The binding affinity was − 4.3 kcal/mol when CVR interacted with the CREBBP. A hydrogen bond was established between CVR with Gln1113 of CREBBP. The CVR/CREBBP binding is promoted by numerous hydrophobic interactions such as with Val1174, Phe1111, Leu1119, Leu1120, Val1115, Pro1114, and Pro1110 of CREBBP. The binding affinity was − 5 kcal/mol when CVR interacted with the HSP90. A hydrogen bond was established between CVR with Asn51 of HSP90. The CVR/HSP90 binding is promoted by numerous hydrophobic interactions such as with Phe138, Leu107, Val150, Val186, Met98, Ala55, Ser52, Thr152, and Thr184 of HSP90. The docking results are summarized in Fig. 4 and Table 3. Also, the proposed mechanism of CVR according to the docking results is illustrated in Fig. 5.

**Table 3:**
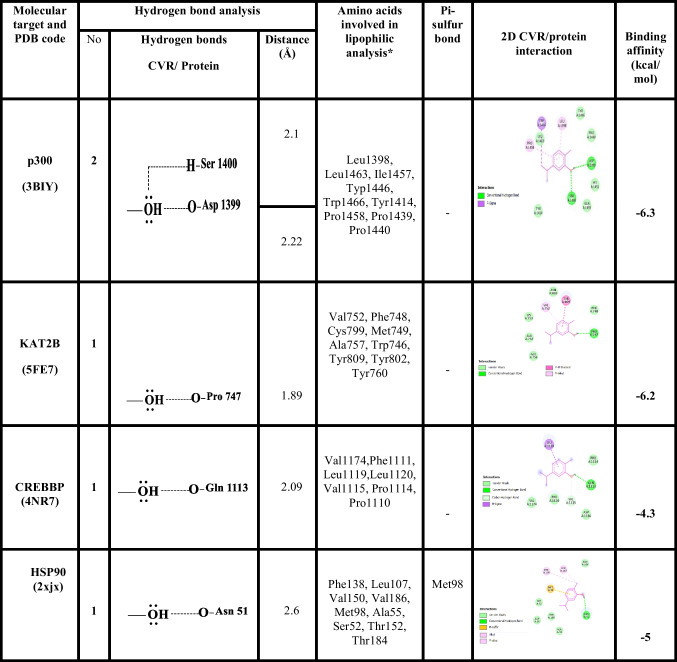
The binding energy, interaction type, and amino acid involved in interaction of p300, KAT2B, CREBBP, and HSP90 with CVR

*CREBBP*, CREB binding protein; *CVR*, carvacrol; *HSP90*, heat shock protein 90; *KAT2B*, lysine acetyltransferase 2B; *p300*, histone acetyltransferase

### Functional enrichment analysis for the studied genes

Functional analyses of the studied genes using DAVID (https://david.ncifcrf.gov/) showed significant enrichment for terms related to cancer, immune system process, cell proliferation, and malignant neoplasm of prostate and breast cancer (Table 4).

**Table 4:** Functional analyses of selected genes

Category	Term	*P*-value	Count	Genes
GAD_DISEASE_CLASS	Cancer	0.015	4	HIF-1α, STAT3, JAK2, FGL1
GOTERM_BP_ALL	Immune system process	0.0039	4	HIF-1α, STAT3, JAK2, FGL1
GOTERM_BP_ALL	Cell proliferation	0.0012	4	HIF-1α, STAT3, JAK2, FGL1
GAD_DISEASE	Type 2 diabetes| edema	0.0049	4	HIF-1α, STAT3, JAK2, FGL1
DISGENET	Prostatic neoplasms	0.004	3	HIF-1α, STAT3, JAK2
DISGENET	Malignant neoplasm of prostate	0.004	3	HIF-1α, STAT3, JAK2
GAD_DISEASE	Breast cancer	0.006	3	HIF-1α, STAT3, JAK2

*FGL1* fibrinogen-like protein 1, *HIF-1α* hypoxia-inducible factor-1 alpha, *JAK2* Janus kinase 2, *STAT3* signal transducers and activators of transduction

### CVR improves liver functions and enhances SOR anti-*cancer* activity

**Fig. 6:**
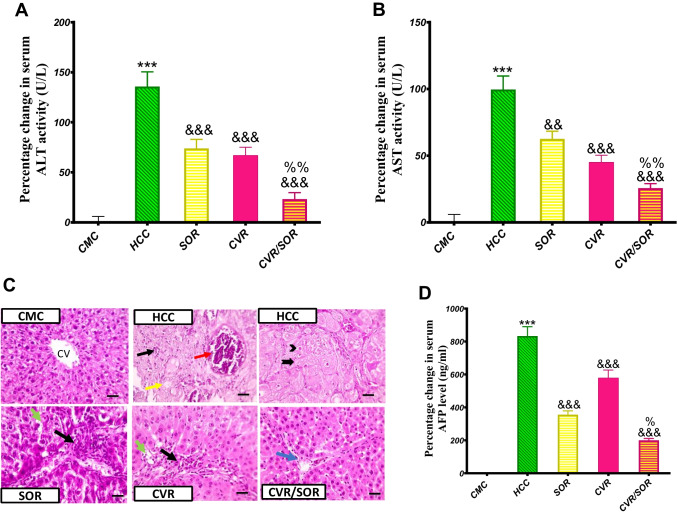
Carvacrol (CVR) improves liver functions and enhances SOR anti-cancer activity. Effect of CVR on hepatocellular carcinoma (HCC) induction was assessed in all groups by the following: **A** measuring serum ALT activity; **B** measuring serum AST activity; **C** histopathological analysis of liver tissue stained with H&E. Yellow arrows illustrate ballooning degeneration; black arrows indicate fibrous septa; red arrows indicate congested blood vessels; blue arrows represent very thin, and short fibrous strands are seen extending from portal areas. Magnification X: 100, bar 100; and **D** measuring serum AFP level. Bars represent mean ± SEM. ^*^Significance against carboxymethyl cellulose (CMC) group (^***^P ≤ 0.0001), ^&^significance against the HCC group (^&&^*P* < 0.01, ^&&&^*P* < 0.001), and ^%^significance against the SOR group (^%%^*P* < 0.01, ^%^*P* < 0.05)

The HCC group showed a significant increase in serum activity of ALT and AST by 135.8% and 174% (*P* ≤ 0.001), respectively, as compared with the CMC group. On the other hand, SOR, CVR, and CVR/SOR treatment significantly suppressed the serum activity of ALT by 26.2%, 29.1%, and 47.7% (*P* ≤ 0.001), respectively, compared with the HCC group. Also, SOR, CVR, and CVR/SOR treatment significantly suppressed the serum activity of AST by 18.6% (*P* ≤ 0.01), 27.3% (*P* ≤ 0.001), and 37.1% (*P* ≤ 0.001), respectively, as compared with the HCC group. Interestingly, CVR/SOR combination showed significant improvement in ALT and AST activities by 29.1% and 22.7% (*P* ≤ 0.01) as compared to the Sora group (Fig. 6A, B).

Liver parts stained with H&E examination showed regular liver structure in the CMC group. The HCC group demonstrated tumor nodules separated from the cirrhotic parenchyma by a thin fibrous capsule and a significant increase in inflammation and degeneration. SOR and CVR groups demonstrated a decrease in fibrosis infiltrated with less leukocytic cell numbers, ballooning degeneration, and/or steatosis. Interestingly, The CVR/SOR group showed greatly improved histology of hepatic parenchyma and retained regular hepatic cord arrangement around central veins and normal sinusoids (Fig. 6C).

The HCC group revealed a significant buildup in serum AFP level by 9.3-fold (*P* < 0.001) as compared with the CMC group, while the daily oral Sora and CARV, for 6 weeks, induced a significant retraction in serum AFP level by 51.2% and 27.3% (*P* < 0.001) as compared with the HCC group, respectively. Also, the CARV/Sora group showed a significant decrease in serum AFP level by 67.9% (*P* < 0.001) and 34.2% (*P* < 0.05) as compared to the HCC and Sora groups, respectively **(**Fig. 6D).

### CVR improves SOR attenuation efficacy against TAA-induced angiogenesis

To further identify CVR as a possible angiogenesis inhibitor in HCC, hepatic HIF-1α level was evaluated.

**Fig. 7:**
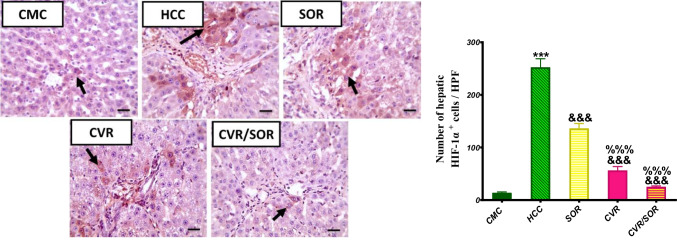
Carvacrol (CVR) increases sorafenib (SOR)’s ability to attenuate thioacetamide (TAA)-caused angiogenesis in rats. The impact of CVR on TAA-induced angiogenesis was evaluated in all groups by assessing the immunohistochemical (IHC) level of the hypoxia-inducible factor-1 alpha (HIF-1α) protein. Black arrows indicate an expression that is positive. Mayer’s hematoxylin was used as a counterstain for IHC. X: 400 bar 50. Bars indicate mean ± SEM. ^*^Significance against the carboxymethyl cellulose (CMC) group (^***^P ≤ 0.0001), ^&^significance against the HCC group (^&&&^*P* ≤ 0.001), and ^%^significance against the SOR group (^%^*P* < 0.05, ^%%%^*P* < 0.001)

The HCC group demonstrated a significant elevation in the hepatic protein expression of HIF-1α by 18.36-fold (*P* ≤ 0.001) as compared to the CMC group. Conversely, HIF-1α protein expression level was significantly decreased in SOR, CVR, and CVR/SOR groups by 1.91-, 5.45-, and 10.52-fold (*P* ≤ 0.001) as compared to the HCC group, respectively. Moreover, HIF-1α protein expression level was markedly decreased in the CVR/SOR group by 5.67-fold (*P* ≤ 0.001) as compared to the SOR group **(**Fig. 7).

### CVR/SOR decreases JAK2, STAT3 gene expressions, and FGLI on gene and protein levels

In order to investigate the fundamental molecular mechanism, CVR effect on the gene expressions of the JAK2/STAT3 pathway, either alone or combined with SOR, was evaluated. Additionally, the influence of this pathway on FGL1 gene expression and protein levels was studied.

**Fig. 8:**
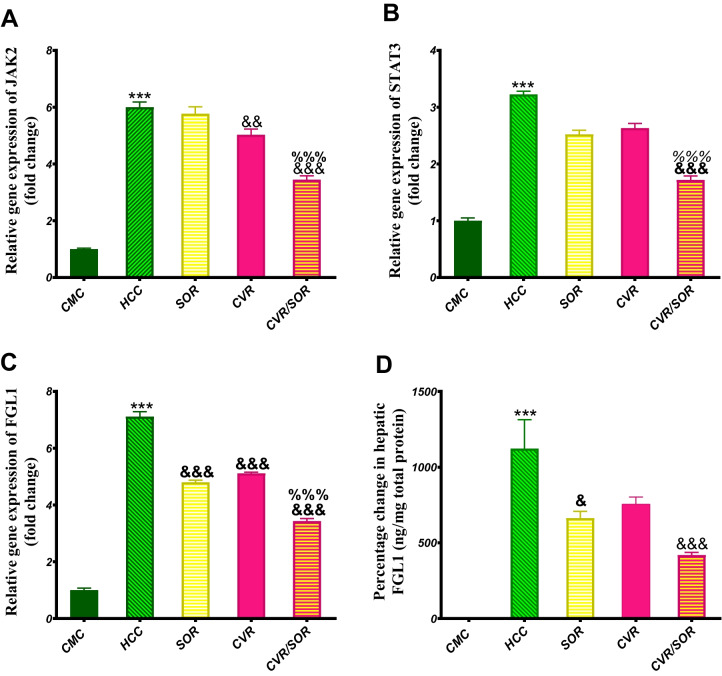
Carvacrol/sorafenib (CVR/SOR) decreases the expression of JAK2 and STAT3 genes as well as FGLI on gene and protein levels. Effect of CVR/SOR was evaluated in all groups by **A** JAK2 relative gene expression by RT-PCR, **B** STAT3 relative gene expression by RT-PCR, **C** FGL1 relative gene expression by RT-PCR, and **D** hepatic FGL1 protein level by ELISA. Bars indicate mean ± SEM. *Significance against the CMC group (***P ≤ 0.0001), ^&^significance against the HCC group (^&^*P* < 0.05; ^&&^*P* < 0.01; ^&&&^*P* < 0.001), and ^%^significance against the SOR group (^%%%^*P* < 0.001). CMC, carboxymethyl cellulose; FGL1, fibrinogen-like protein 1; HIF-1α, hypoxia-inducible factor-1 alpha; JAK2, Janus kinase 2; STAT3, signal transducers and activators of transduction

The HCC group demonstrated a significant elevation in JAK2 and STAT3 gene expression by 6- and 3.2-fold (*P* ≤ 0.001) as compared to the CMC group, respectively. On the other hand, the JAK2 and STAT3 expression levels were significantly decreased in the CVR/SOR group by 1.69- and 1.9-fold (*P* ≤ 0.001) as compared with the SOR group, respectively (Fig. 8A, B).

Furthermore, the HCC group demonstrated a significant increase in FGL1 on gene expression and protein level by 7.1- and 12.2-fold (*P* ≤ 0.001) as compared with the CMC group, respectively. On the other hand, FGL1 gene expression and protein level were significantly decreased in the SOR group by 1.48 (*P* ≤ 0.001) and 1.63 (*P* < 0.05) fold, respectively, compared with the HCC group. Also, CVR/SOR groups showed a significant decrease in FGL1 gene and protein expression by 2.1- and 2.37-fold (*P* ≤ 0.0001) as compared with the HCC group, respectively. It is noteworthy that FGL1 gene expression and protein level were significantly decreased in the CVR/SOR group by 1.4- and 1.48-fold (*P* ≤ 0.001) as compared to the SOR group, respectively (Fig. 8C, D).

### CVR potentiates SOR-induced tumor immunity reactivation

**Fig. 9:**
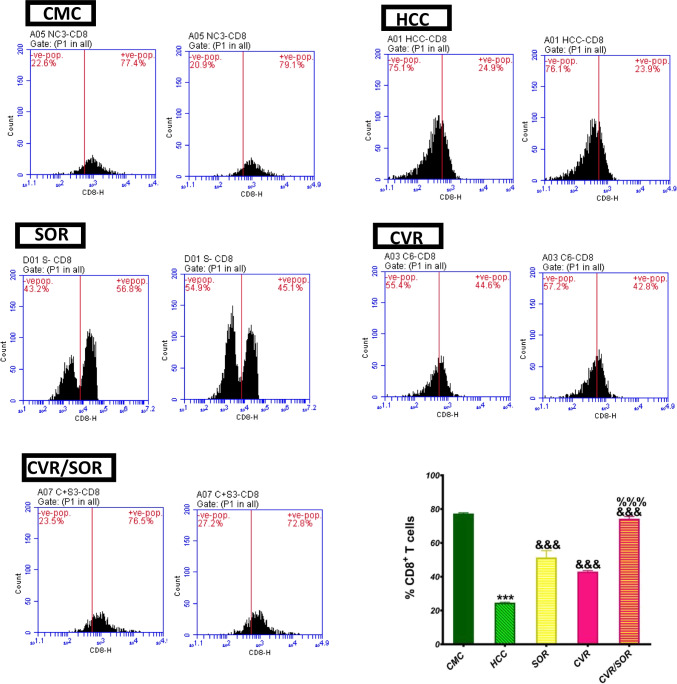
Carvacrol (CVR) potentiates sorafenib (SOR) caused the reactivation of tumor immunity in rats. Tumor immunity in all groups was assessed by calculating the proportion of CD8^+^ T cells using flow cytometry. Bars indicate mean ± SEM. *Significance against the CMC group (***P ≤ 0.0001), ^&^significance against the HCC group (^&&&^*P* ≤ 0.0001), and ^%^significance against the SOR group (^%%%^*P* < 0.001). CMC, carboxymethyl cellulose; HCC, hepatocellular

To study the CVR effect, either alone or combined with SOR, on the antitumor response of the immune system, the CD8^+^ T cell number was measured using flow cytometry. The analysis of intrahepatic cells revealed that the frequency of CD8^+^ T cells was 3.2-fold (*P* ≤ 0.001) lower in the HCC group compared to the CMC group, whereas it was increased by 2-, 1.75-, and 3-fold (*P* ≤ 0.001) in rats treated with SOR, CVR, and CVR/SOR therapies compared to the HCC group, respectively. As well, the frequency of CD8^+^ T cells was raised by 1.45 (*P* ≤ 0.001) in rats treated with CVR/SOR combination treatment as compared to the SOR group (Fig. 9).

### HIF-1α downregulation induced by SOR, CVR, and their combination correlates with STAT3 and FGL1 downregulation

**Fig. 10:**
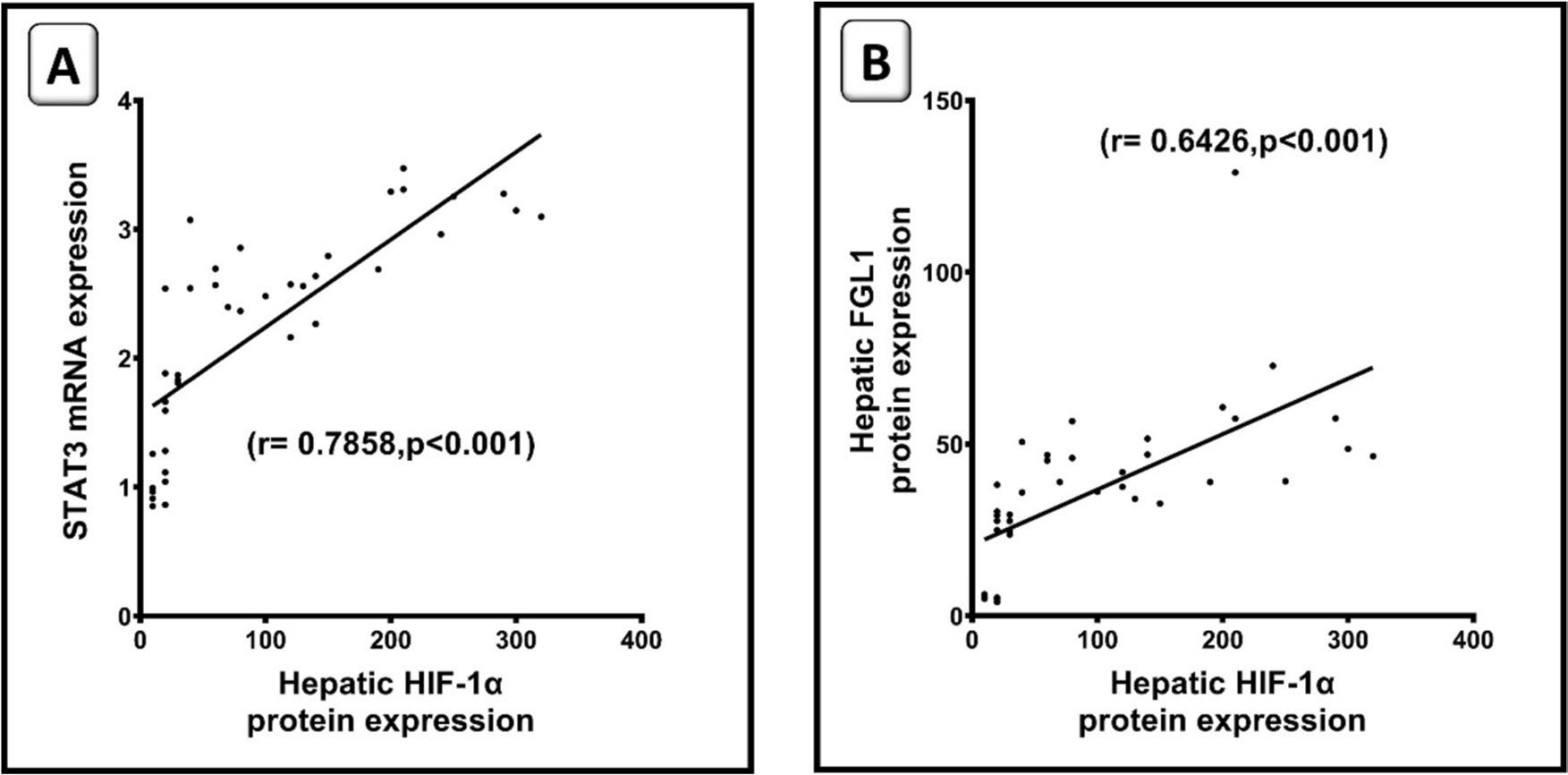
Hypoxia-inducible factor-1 alpha (HIF-1α) downregulation induced by sorafenib (SOR), carvacrol (CVR), and their combination correlates with signal transducers and activators of transduction (STAT3) and fibrinogen-like protein 1 **(**FGL1) downregulation. **A** The expression of HIF-1α is positively correlated with STAT3 gene expression. **B** The expression of HIF-1α is positively correlated with FGL1 protein expression

As far as HIF-1α level is a downstream effector gene of STAT3 and hypoxia suppress tumor microenvironment immunity, it was interesting to analyze the correlations between HIF-1α with either STAT3 or FGL1 in HCC. Statistical analysis demonstrated that there is a significant direct correlation between HIF-1α and STAT3 (*r* = 0.78) (Fig. 10A) and a significant direct correlation between HIF-1α and FGL1 (*r* = 0.64) (Fig. 10B).

## Discussion

HCC is a malignancy characterized by overgrowth of tumor cells and increasing oxygen demand, leading to the development of a hypoxic microenvironment (Guo et al. 2020). Also, Hypoxia is implicated in all HCC development stages. In early stages, HIFs in Kupffer cells stimulate liver cirrhosis by stimulating hepatic stellate cells to generate profibrogenic factors, while in later stages, HIF function as master regulators to hinder the innate immunity allowing cancer cells to evade immune surveillance (Yuen and Wong 2020). In hypoxic condition, HIF-1α protein expression is reported to be markedly increased and stabilized and to be responsible for HCC progression (Feng et al. 2018). Hypoxia and overexpression of HIF-1α have been intrinsically linked to treatment failure, a higher risk of invasion, metastasis, tumor immunity evasion, and tumor aggressiveness (Rath et al. 2022; Wang et al. 2022a; Chen et al. 2023). Thus, finding compounds that inhibit HIF activation may have a great potential for HCC treatment.

The hypoxic conditions within tumors lead to a marked increase and stabilization of HIF-1α protein expression, which is closely associated with HCC progression (Huynh et al. 2023). Our *in silico* study showed that HIF-1α was highly expressed in HCC tissues and correlated with histological subtypes and individual cancer stages. Patients with high HIF-1α expression showed worse OS and PFS and higher RFS than those with low HIF-1α expression. Also, TAA-caused HCC *in vivo* induced the elevation of HIF-1α protein expression. Those results came in accordance with previous studies analyzing the clinicopathological characteristics of HCC patients that reported overexpression of HIF-1α correlated with tumor-to-lymph node metastasis stage III, Barcelona clinical hepatocellular carcinoma (BCLC) stage C (Cheng et al. 2019), vascular invasion, tumor size, and portal vein tumor thromboembolism (Wang et al. 2018; Méndez-Blanco et al. 2021). The survival analysis showed that HCC patients with high HIF-1α mRNA expression had poor overall survival (Wang et al. 2018; Cheng et al. 2019), disease-free survival (Qian et al. 2021), and prognosis (Ding et al. 2021; Qian et al. 2021).

The interplay between HIF-1α and reduced SOR efficiency has been well documented. El Shorbagy et al. (2021) found that patients who had high tissue HIF-1α expression had considerably worse SOR response and survival than those who had low expression (El Shorbagy et al. 2021). Song et al. (2019) have reported that HIF-1α limits the efficiency of SOR in HCC cells (Song et al. 2019). In accordance with that, Liang et al. (2013) found that the efficacy of SOR in HCC cells improved when combined with curcumin analog EF24 by promoting the proteasome degradation of the VHL-dependent HIF-1α (Liang et al. 2013). In addition, the inhibition of USP14, a HIF-1α inducer, has been stated to enhance the sensitivity to SOR (Lv et al. 2021). In SOR-resistant HCC cells and HCC xenograft animal models, it was stated that the use of genistein, a natural isoflavone, increased the anti-cancer impact of SOR by decreasing HIF-1α and inducing mitochondrial apoptosis (Li et al. 2017). Also, simvastatin decreased proliferation, increased apoptosis, and re-sensitized HCC cells to SOR by inhibiting the HIF-1α/PPAR-γ/PKM2 axis (Feng et al. 2020). In the present study, the CVR-treated group showed a significant decrease in the expression of HIF-1α protein that has an important role in its antitumor efficacy as it significantly reduced liver damage, serum ALT, AST activities, and AFP level. This result is in agreement with Ahmed et al. (2013) who demonstrated that rats exposed to N-nitrosodiethylamine and treated with CVR show an improved AFP serum level (Ahmed et al. 2013). Similarly, other studies have shown that CVR-containing extracts, such as Thymus vulgaris, can decrease AFP levels and suppress tumor growth (Nickavar et al. 2005; Hamzawy et al. 2012). Moreover, Yin et al. (2012) reported the ability of CVR to suppress the growth and proliferation of HepG2 by reducing phosphorylated ERK1/2 (Yin et al. 2012) which is implicated in the regulation of HIF-1α activity (Saber et al. 2021). Based on this, the capability of CVR to enhance SOR antitumor activity may be attributed to its ability to inhibit HIF-1α.

The JAK2/STAT3 pathway is known to regulate the expression and function of a variety of genes that are critical to immune evasion (Liu et al. 2018; Wu et al. 2023). In HCC, aberrant hyperactivation of JAK2/STAT3 signaling has been linked to immune evasion and chemotherapy resistance (Hin Tang et al. 2020; Liu et al. 2023)(Li et al. 2020; Zhang et al. 2020). One of the STAT3 targeted genes is FGL1 (Wang et al. 2020; Yousef et al. 2023b). FGL1 is a lymphocyte activation gene-3 (LAG-3) inhibitory ligand and immune suppressant molecule. Hepatocytes release FGL1 as a mitogen to encourage hepatocyte growth. FGL1 is typically low in healthy hepatocytes, but it is dramatically elevated in HCC cells (Jin et al. 2022). It has also been found that FGL1 inhibits tumor cell-specific T lymphocyte activation and their identification of antigens (Kutoka et al. 2022). On the other hand, CD8^+^ cytotoxic T lymphocyte-induced antitumor immunity is supported by FGL1 silencing or blocking (Hossain et al. 2021). Also, Salman et al. (2022) found that systemic HIF inhibition in HCC increases the expression of CXCL9 and CXCL10 and relieves immunosuppression by enhancing CD8^+^ T cells and NK cells recruitment to the tumor (Salman et al. 2022). In agreement with previously published studies, the present study showed an increase in FGL1 gene and protein expression and a decrease in the number of CD8^+^ T cells in the HCC group. On the other hand, the inhibition of HIF-1α by CVR improved SOR-caused FGL1 downregulation and increased the CD8^+^ T cells number.

Numerous studies reported the strong relationship between immune evasion and intratumoral hypoxia. HIF-1α has been linked to the increase in plasmacytoid dendritic cells (Pang et al. 2021) and myeloid-derived suppressor cells (MDSCs) (Chiu et al. 2017; Hinshaw and Shevde 2019), tumor-associated macrophages (TAM) (Kumar and Gabrilovich 2014), as well as to the induction of IL-1β (Zhang et al. 2018; He et al. 2021; Kiss et al. 2021) expression in the TME, which results in immune escape. Hypoxic zones within tumors in mouse models were shown to be devoid of T lymphocytes (Jayaprakash et al. 2018). In hypoxic tumor cells, HIF-1α was reported to transactivate CD274, the gene encoding PD-L1 protein, allowing tumor immune escape from CD8^+^ cytotoxic T cells (Barsoum et al. 2014). Hypoxia increases PD-L1 in an HIF-1-dependent manner and inhibits T cell activation (Noman et al. 2014). SLC7A11 has been reported to activate the HIF-1 cascade to increase PD-L1 expression and leads to the development of an immunosuppressive milieu, which encourages the HCC metastasis (He et al. 2021). Reportedly, circPRDM4 mostly promoted immune evasion of HCC cells through the HIF-1/PD-L1 axis (Chen et al. 2023). In agreement with previous studies, our results proved that HIF-1α expression positively correlated with STAT3 and FGL1.

The limitations of this study include the necessity for subsequent clinical trials to validate our in silico and in vivo findings, as well as to assess the clinical applicability of the CVR/SOR combination in the treatment of HCC. Also, while our study provided valuable insights into CD8^+^ T cell abundance, we acknowledge that a more comprehensive assessment of cell-mediated immunity would require analyzing additional parameters. Future studies could focus on measuring CD8^+^ T cell activation markers, such as CD69 and CD25, to gain a deeper understanding of the functional state of these cells.

## Conclusion

**Fig. 11:**
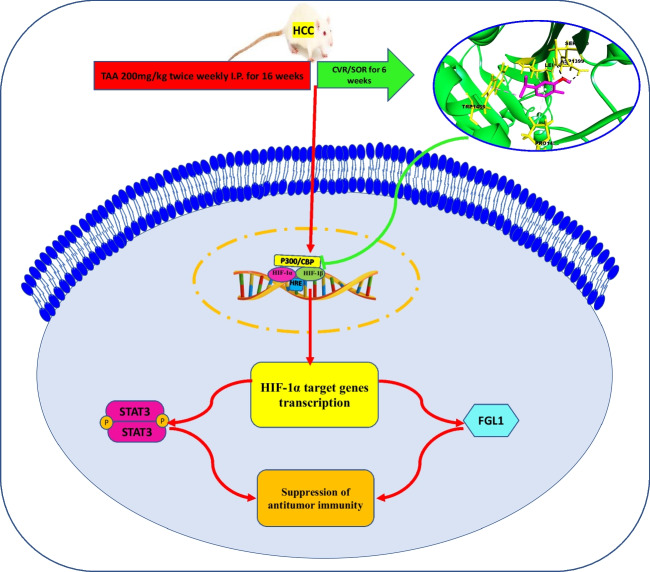
Proposed mechanism of CVR/SOR anti-HCC activity. CVR/SOR combination modulates anti-HCC immunity through modulating HIF-1α/JAK2/STAT3/FGL1 pathway. CVR, carvacrol; FGL1, fibrinogen-like protein 1; HIF-1α, hypoxia-inducible factor-1 alpha; JAK2, Janus kinase 2; SOR, sorafenib; STAT3, signal transducers and activators of transduction; TAA, thioacetamide

Through in silico and in vivo experiments, we demonstrated the effectiveness of CVR in enhancing the antitumor response of HCC to SOR. As a result, CVR has been suggested as a potential treatment for HCC, probably through suppressing HIF-1α. Up to our knowledge, this is the first study to report the effect of CVR on FGL1. In addition, this research supports the possibility of a unique therapeutic use of the CVR/SOR combination for modulating antitumor immunity through modulating HIF-1α/JAK2/STAT3/FGL1 pathway (Fig. 11). Thus, this study could serve as a base for advanced investigations, preferably large-scale validation in clinical trials to evaluate the antitumor effect of CVR in HCC patients receiving SOR.

## Supplementary Information

Below is the link to the electronic supplementary material.

Supplementary file1 (PPTX 47.6 KB)

Supplementary file2 (PDF 34.8 KB)

## Data Availability

Data are available upon request.

## Abbreviations

AFP: Alpha fetoprotein
ALT: Alanine aminotransferase
AST: Aspartate aminotransferase
BCLC: Barcelona clinical hepatocellular carcinoma
CMC: Carboxymethyl cellulose
CVR: Carvacrol
DSS: Disease-specific survival
ELISA: Enzyme-linked immunosorbent assay
FGL1: Fibrinogen-like protein 1
FIH: Factor inhibiting HIF-1
GAPDH: Glyceraldehyde-3-phosphate dehydrogenase
H&E: Hematoxylin and eosin
HCC: Hepatocellular carcinoma
HIF-1α: Hypoxia-inducible factor 1-alpha
HRE: Hypoxia response element
HSP90: Heat shock protein 90
i.p.: Intraperitoneal
IHC: Immunohistochemistry
JAK: Janus kinase
KM: Kaplan-Meier
LAG-3: Lymphocyte activation gene-3
MDSCs: Myeloid-derived suppressor cells
MNA: Multilevel neighbors of atoms
NIH: National Institutes of Health
OS: Overall survival
Pa: Probability to be active
PBS: Phosphate buffer saline
PFS: Progression-free survival
PHD: Prolyl-4-hydroxylase
Pi: Probability to be inactive
qRT-PCR: Quantitative real-time polymerase chain reaction
RFS: Relapse-free survival
SOR: Sorafenib
STAT: Signal transducers and activators of transduction
TAA: Thioacetamide
TAM: Tumor-associated macrophages
TCGA: The Cancer Genome Atlas
TISIDB: Tumor-Immune System Interaction Database
VHL: Von Hippel-Lindau
