# Supplementary material for: Carvacrol potentiates immunity and sorafenib anti-cancer efficacy by targeting HIF-1α/STAT3/ FGL1 pathway: in silico and in vivo study
Source: Naunyn Schmiedebergs Arch Pharmacol. 2024 Oct 28;398(4):4335–53. doi: 10.1007/s00210-024-03530-9 (PMC11978551; doi:10.1007/s00210-024-03530-9)
Supplement: Supplementary file 1 — Supplementary file1 (PPTX 47.6 KB) [file 210_2024_3530_MOESM1_ESM.pptx]

## Slide 1
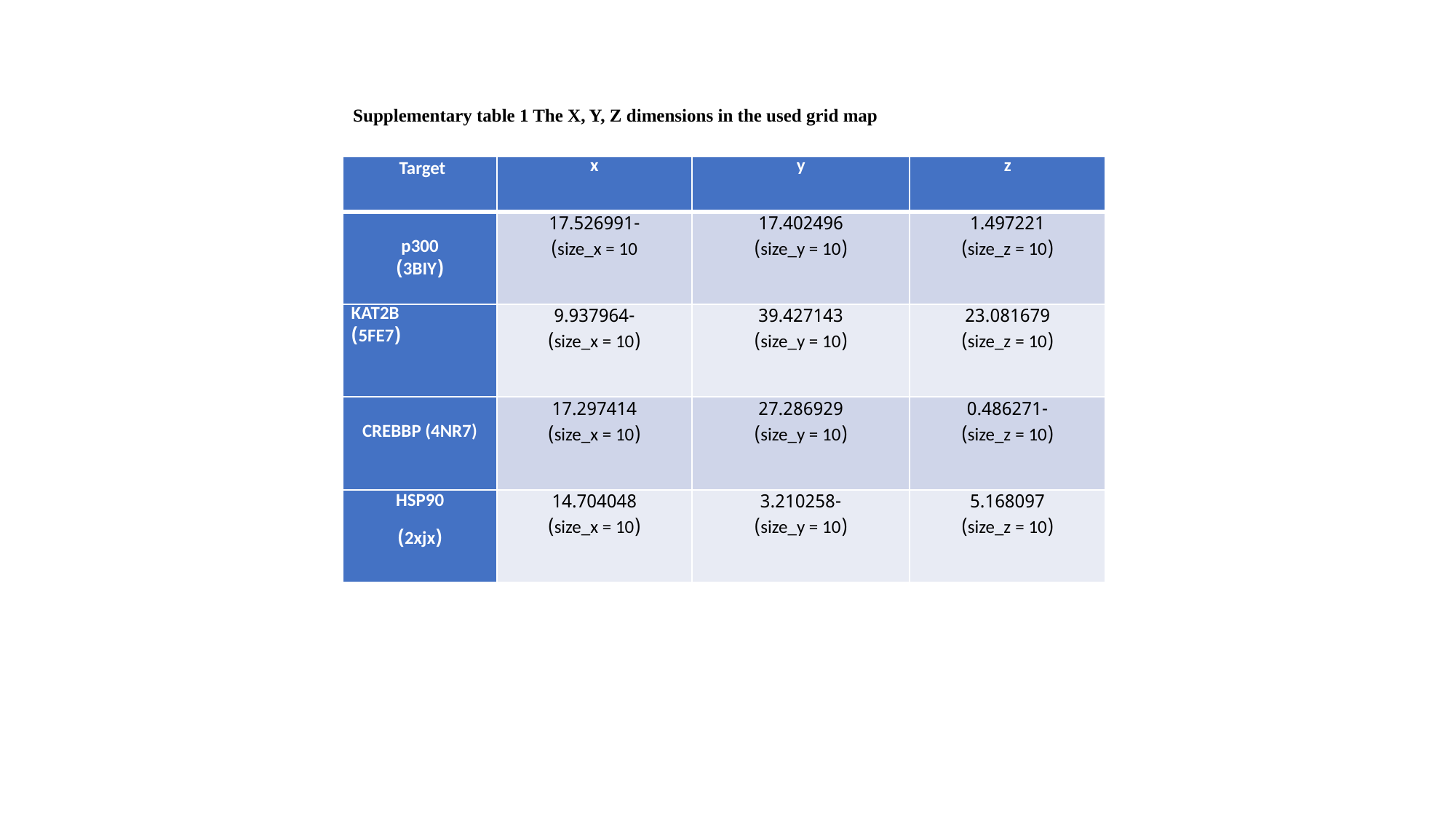

Supplementary table 1 The X, Y, Z dimensions in the used grid map
| Target | x | y | z |
| --- | --- | --- | --- |
| p300 (3BIY) | -17.526991 size\_x = 10) | 17.402496 (size\_y = 10) | 1.497221 (size\_z = 10) |
| KAT2B (5FE7) | -9.937964 (size\_x = 10) | 39.427143 (size\_y = 10) | 23.081679 (size\_z = 10) |
| CREBBP (4NR7) | 17.297414 (size\_x = 10) | 27.286929 (size\_y = 10) | -0.486271 (size\_z = 10) |
| HSP90 (2xjx) | 14.704048 (size\_x = 10) | -3.210258 (size\_y = 10) | 5.168097 (size\_z = 10) |

## Slide 2
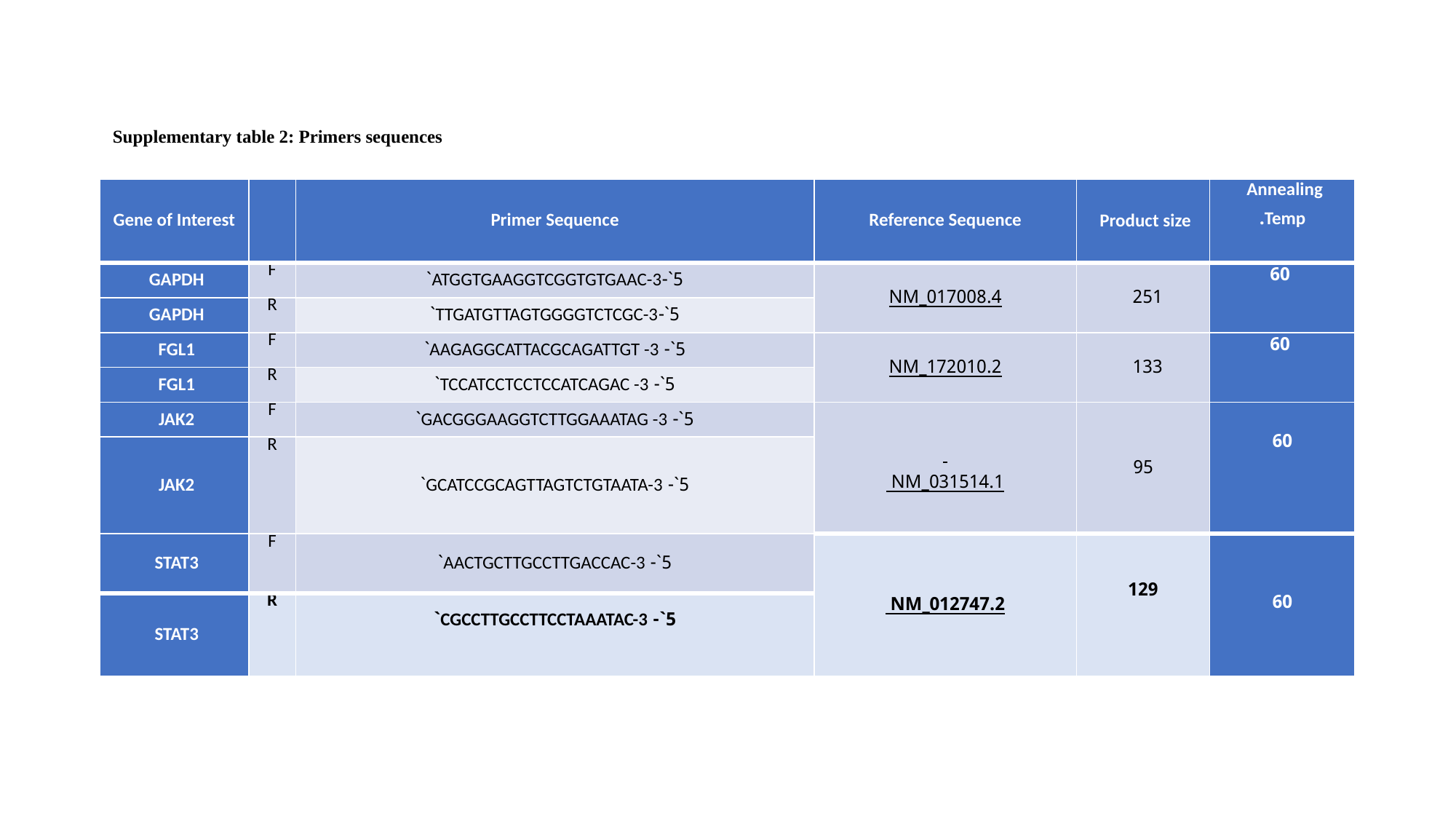

Supplementary table 2: Primers sequences
| Gene of Interest | | Primer Sequence | Reference Sequence | Product size | Annealing Temp. |
| --- | --- | --- | --- | --- | --- |
| GAPDH | F | 5`-ATGGTGAAGGTCGGTGTGAAC-3` | NM\_017008.4 | 251 | 60 |
| GAPDH | R | 5`-TTGATGTTAGTGGGGTCTCGC-3` | | | |
| FGL1 | F | 5`- AAGAGGCATTACGCAGATTGT -3` | NM\_172010.2 | 133 | 60 |
| FGL1 | R | 5`- TCCATCCTCCTCCATCAGAC -3` | | | |
| JAK2 | F | 5`- GACGGGAAGGTCTTGGAAATAG -3` | NM\_031514.1 | 95 | 60 |
| JAK2 | R | 5`- GCATCCGCAGTTAGTCTGTAATA-3` | | | |
| STAT3 | F | 5`- AACTGCTTGCCTTGACCAC-3` | NM\_012747.2 | 129 | 60 |
| STAT3 | R | 5`- CGCCTTGCCTTCCTAAATAC-3` | | | |
