# Supplementary material for: Carvacrol potentiates immunity and sorafenib anti-cancer efficacy by targeting HIF-1α/STAT3/ FGL1 pathway: in silico and in vivo study
Source: Naunyn Schmiedebergs Arch Pharmacol. 2024 Oct 28;398(4):4335–53. doi: 10.1007/s00210-024-03530-9 (PMC11978551; doi:10.1007/s00210-024-03530-9)
Supplement: Supplementary file 2 — Supplementary file2 (PDF 34.8 KB) [file 210_2024_3530_MOESM2_ESM.pdf]

SwissTargetPrediction

Target	Common name	Uniprot ID	ChEMBL ID	Target Class	Probability*	Known actives (3D/2D)
Cyclooxygenase-1	PTGS1	P23219	CHEMBL221	Oxidoreductase	0.936391357275	28 / 10
Transient receptor potential cation channel subfamily A member 1	TRPA1	O75762	CHEMBL6007	Voltage-gated ion channel	0.254594304531	5 / 1
Serotonin 2b (5-HT2b) receptor	HTR2B	P41595	CHEMBL1833	Family A G protein-coupled receptor	0.053517944289	8 / 3
Carbonic anhydrase II	CA2	P00918	CHEMBL205	Lyase	0.053517944289	74 / 17
Histone deacetylase 6	HDAC6	Q9UBN7	CHEMBL1865	Eraser	0.053517944289	5 / 0
Carbonic anhydrase IV	CA4	P22748	CHEMBL3729	Lyase	0.053517944289	4 / 2
Androgen Receptor	AR	P10275	CHEMBL1871	Nuclear receptor	0.0439186325197	20 / 9
Serum albumin	ALB	P02768	CHEMBL3253	Secreted protein	0.0439186325197	0 / 5
Cytochrome P450 19A1	CYP19A1	P11511	CHEMBL1978	Cytochrome P450	0.0439186325197	45 / 14
Tyrosine-protein kinase JAK1	JAK1	P23458	CHEMBL2835	Kinase	0.0439186325197	14 / 0
Tyrosine-protein kinase JAK2	JAK2	O60674	CHEMBL2971	Kinase	0.0439186325197	29 / 0
Protein kinase C alpha	PRKCA	P17252	CHEMBL299	Kinase	0.0439186325197	2 / 0
Serine/threonine-protein kinase Aurora-A	AURKA	O14965	CHEMBL4722	Kinase	0.0439186325197	5 / 0
Dopamine transporter	SLC6A3	Q01959	CHEMBL238	Electrochemical transporter	0.0439186325197	4 / 12
Tyrosinase	TYR	P14679	CHEMBL1973	Oxidoreductase	0.0439186325197	2 / 8
Tyrosine-protein kinase receptor FLT3	FLT3	P36888	CHEMBL1974	Kinase	0.0439186325197	5 / 0
Dopamine D5 receptor	DRD5	P21918	CHEMBL1850	Family A G protein-coupled receptor	0.0439186325197	0 / 2
Dopamine D3 receptor	DRD3	P35462	CHEMBL234	Family A G protein-coupled receptor	0.0439186325197	0 / 15
Histone deacetylase 8	HDAC8	Q9BY41	CHEMBL3192	Eraser	0.0439186325197	9 / 4
11-beta-hydroxysteroid dehydrogenase 1	HSD11B1	P28845	CHEMBL4235	Enzyme	0.0439186325197	30 / 0
Indoleamine 2,3-dioxygenase	IDO1	P14902	CHEMBL4685	Enzyme	0.0439186325197	12 / 0
Serotonin 6 (5-HT6) receptor	HTR6	P50406	CHEMBL3371	Family A G protein-coupled receptor	0.0439186325197	0 / 2
Estrogen-related receptor gamma	ESRRG	P62508	CHEMBL4245	Nuclear receptor	0.0439186325197	0 / 1
Nitric-oxide synthase, brain	NOS1	P29475	CHEMBL3568	Enzyme	0.0439186325197	14 / 0
Nitric-oxide synthase, endothelial	NOS3	P29474	CHEMBL4803	Enzyme	0.0439186325197	8 / 0
Muscarinic	CHRM2	P08172	CHEMBL211	Family A G	0.0439186325197	3 / 1

Target	Common name	Uniprot ID	ChEMBL ID	Target Class	Probability*	Known actives (3D/2D)
acetylcholine receptor M2				protein-coupled receptor		
Muscarinic acetylcholine receptor M1	CHRM1	P11229	CHEMBL216	Family A G protein-coupled receptor	0.0439186325197	3 / 1
Acid ceramidase	ASAH1	Q13510	CHEMBL5463	Enzyme	0.0	8 / 0
Alkaline phosphatase, tissue-nonspecific isozyme	ALPL	P05186	CHEMBL5979	Enzyme	0.0	5 / 0
Melanocortin receptor 4	MC4R	P32245	CHEMBL259	Family A G protein-coupled receptor	0.0	1 / 0
c-Jun N-terminal kinase 3	MAPK10	P53779	CHEMBL2637	Kinase	0.0	6 / 0
Microtubule-associated protein tau	MAPT	P10636	CHEMBL1293224	Unclassified protein	0.0	0 / 1
Microtubule-associated protein 2	MAP2	P11137	CHEMBL2390810	Unclassified protein	0.0	7 / 0
GABA-A receptor; alpha-1/beta-2/ gamma-2	GABRA1 GABRB2 GABRG2	P14867 P47870 P18507	CHEMBL2095172	Ligand-gated ion channel	0.0	3 / 3
c-Jun N-terminal kinase 1	MAPK8	P45983	CHEMBL2276	Kinase	0.0	7 / 0
c-Jun N-terminal kinase 2	MAPK9	P45984	CHEMBL4179	Kinase	0.0	3 / 0
NAD-dependent deacetylase sirtuin 2	SIRT2	Q8IXJ6	CHEMBL4462	Eraser	0.0	12 / 0
Serotonin 2c (5-HT2c) receptor	HTR2C	P28335	CHEMBL225	Family A G protein-coupled receptor	0.0	9 / 3
Norepinephrine transporter	SLC6A2	P23975	CHEMBL222	Electrochemical transporter	0.0	3 / 4
Arachidonate 12-lipoxygenase	ALOX12	P18054	CHEMBL3687	Enzyme	0.0	0 / 7
Leukotriene B4 receptor 1	LTB4R	Q15722	CHEMBL3911	Family A G protein-coupled receptor	0.0	0 / 1
Dual specificty protein kinase CLK1	CLK1	P49759	CHEMBL4224	Kinase	0.0	0 / 1
DNA polymerase beta (by homology)	POLB	P06746	CHEMBL2392	Enzyme	0.0	0 / 3
Cannabinoid receptor 1	CNR1	P21554	CHEMBL218	Family A G protein-coupled receptor	0.0	9 / 34
Calcium-activated potassium channel subunit alpha-1	KCNMA1	Q12791	CHEMBL4304	Voltage-gated ion channel	0.0	7 / 0
Glyceraldehyde-3-phosphate dehydrogenase liver	GAPDH	P04406	CHEMBL2284	Oxidoreductase	0.0	0 / 1
Histone deacetylase 3	HDAC3	O15379	CHEMBL1829	Eraser	0.0	3 / 0
Tubulin beta-1 chain	TUBB1	Q9H4B7	CHEMBL1915	Structural protein	0.0	3 / 0

Target	Common name	Uniprot ID	ChEMBL ID	Target Class	Probability*	Known actives (3D/2D)
Histone deacetylase 1	HDAC1	Q13547	CHEMBL325	Eraser	0.0	7 / 0
Histone deacetylase 11	HDAC11	Q96DB2	CHEMBL3310	Eraser	0.0	2 / 0
Histone deacetylase 10	HDAC10	Q969S8	CHEMBL5103	Eraser	0.0	2 / 0
Dopamine D1 receptor	DRD1	P21728	CHEMBL2056	Family A G protein-coupled receptor	0.0	8 / 2
Dual specificity protein phosphatase 3	DUSP3	P51452	CHEMBL2635	Phosphatase	0.0	4 / 0
Acetylcholinesterase	ACHE	P22303	CHEMBL220	Hydrolase	0.0	10 / 8
G-protein coupled receptor 84	GPR84	Q9NQS5	CHEMBL3714079	Family A G protein-coupled receptor	0.0	8 / 0
Serine/threonine-protein kinase AKT	AKT1	P31749	CHEMBL4282	Kinase	0.0	7 / 0
Tyrosine-protein kinase JAK3	JAK3	P52333	CHEMBL2148	Kinase	0.0	15 / 0
Epoxide hydratase	EPHX2	P34913	CHEMBL2409	Protease	0.0	30 / 0
Lysine-specific demethylase 4D-like	KDM4E	B2RXH2	CHEMBL1293226	Eraser	0.0	1 / 2
D-amino-acid oxidase	DAO	P14920	CHEMBL5485	Enzyme	0.0	6 / 0
Nischarin	NISCH	Q9Y2I1	CHEMBL3923	Other cytosolic protein	0.0	27 / 0
Estradiol 17-beta-dehydrogenase 3	HSD17B3	P37058	CHEMBL4234	Enzyme	0.0	4 / 0
Dopamine D2 receptor	DRD2	P14416	CHEMBL217	Family A G protein-coupled receptor	0.0	6 / 43
Dopamine D4 receptor	DRD4	P21917	CHEMBL219	Family A G protein-coupled receptor	0.0	1 / 5
Cytochrome P450 11B1	CYP11B1	P15538	CHEMBL1908	Cytochrome P450	0.0	10 / 0
Troponin, cardiac muscle	TNNC1 TNNT2 TNNI3	P63316 P45379 P19429	CHEMBL2095202	Unclassified protein	0.0	1 / 0
Adrenergic receptor beta	ADRB2	P07550	CHEMBL210	Family A G protein-coupled receptor	0.0	0 / 21
Beta-1 adrenergic receptor (by homology)	ADRB1	P08588	CHEMBL213	Family A G protein-coupled receptor	0.0	0 / 8
Cyclin-dependent kinase 2/cyclin A	CDK2 CCNA1 CCNA2	P24941 P78396 P20248	CHEMBL2094128	Other cytosolic protein	0.0	3 / 0
Interleukin-8 receptor B	CXCR2	P25025	CHEMBL2434	Family A G protein-coupled receptor	0.0	4 / 0
Cyclin-dependent kinase 2	CDK2	P24941	CHEMBL301	Kinase	0.0	6 / 0
Tyrosine-protein kinase BRK	PTK6	Q13882	CHEMBL4601	Kinase	0.0	6 / 0

Target	Common name	Uniprot ID	ChEMBL ID	Target Class	Probability*	Known actives (3D/2D)
Muscarinic acetylcholine receptor M5	CHRM5	P08912	CHEMBL2035	Family A G protein-coupled receptor	0.0	1 / 0
C-C chemokine receptor type 2	CCR2	P41597	CHEMBL4015	Family A G protein-coupled receptor	0.0	2 / 0
Estrogen receptor beta	ESR2	Q92731	CHEMBL242	Nuclear receptor	0.0	24 / 66
Pantothenate kinase 3	PANK3	Q9H999	CHEMBL3407328	Enzyme	0.0	1 / 0
Aldehyde dehydrogenase 1A1 (by homology)	ALDH1A1	P00352	CHEMBL3577	Enzyme	0.0	1 / 0
Histamine H4 receptor	HRH4	Q9H3N8	CHEMBL3759	Family A G protein-coupled receptor	0.0	30 / 0
L-lactate dehydrogenase A chain	LDHA	P00338	CHEMBL4835	Enzyme	0.0	0 / 12
L-lactate dehydrogenase B chain	LDHB	P07195	CHEMBL4940	Enzyme	0.0	0 / 10
Cathepsin K	CTSK	P43235	CHEMBL268	Protease	0.0	11 / 0
Cytochrome P450 26B1	CYP26B1	Q9NR63	CHEMBL3713687	Cytochrome P450	0.0	1 / 0
Carbonic anhydrase VA	CA5A	P35218	CHEMBL4789	Lyase	0.0	7 / 0
Cytochrome P450 26A1	CYP26A1	O43174	CHEMBL5141	Cytochrome P450	0.0	1 / 0
Glutaminyl-peptide cyclotransferase	QPCT	Q16769	CHEMBL4508	Enzyme	0.0	14 / 0
Alpha-1b adrenergic receptor	ADRA1B	P35368	CHEMBL232	Family A G protein-coupled receptor	0.0	0 / 2
Monoamine oxidase A	MAOA	P21397	CHEMBL1951	Oxidoreductase	0.0	6 / 2
Serotonin transporter	SLC6A4	P31645	CHEMBL228	Electrochemical transporter	0.0	4 / 6
Serine/threonine-protein kinase PAK 1	PAK1	Q13153	CHEMBL4600	Kinase	0.0	1 / 0
GABA-B receptor	GABBR2 GABBR1	O75899 Q9UBS5	CHEMBL2111463	Family C G protein-coupled receptor	0.0	0 / 1
Epoxide hydrolase 1	EPHX1	P07099	CHEMBL1968	Protease	0.0	10 / 0
Progesterone receptor	PGR	P06401	CHEMBL208	Nuclear receptor	0.0	24 / 0
G-protein coupled receptor 55	GPR55	Q9Y2T6	CHEMBL1075322	Family A G protein-coupled receptor	0.0	0 / 1
N-arachidonyl glycine receptor	GPR18	Q14330	CHEMBL2384898	Family A G protein-coupled receptor	0.0	0 / 1
Ribonuclease H1	RNASEH1	O60930	CHEMBL5893	Enzyme	0.0	0 / 1
Thrombin	F2	P00734	CHEMBL204	Protease	0.0	2 / 0
Fibroblast growth factor receptor 1	FGFR1	P11362	CHEMBL3650	Kinase	0.0	6 / 0

Target	Common name	Uniprot ID	ChEMBL ID	Target Class	Probability*	Known actives (3D/2D)
Lymphocyte differentiation antigen CD38	CD38	P28907	CHEMBL4660	Enzyme	0.0	11 / 0
Estrogen receptor alpha	ESR1	P03372	CHEMBL206	Nuclear receptor	0.0	22 / 63
Butyrylcholinesterase	BCHE	P06276	CHEMBL1914	Hydrolase	0.0	3 / 0
